# Supplementary material for: Muc16CD is a novel CAR T cell target antigen for the treatment of pancreatic cancer
Source: Mol Ther Oncol. 2024 Sep 2;32(4):200868. doi: 10.1016/j.omton.2024.200868 (PMC11426034; doi:10.1016/j.omton.2024.200868)
Supplement: Document S1. Figures S1–S3 [file mmc1.pdf]

OMTON, Volume *32*

Supplemental information

Muc16CD is a novel CAR T cell target antigen for the treatment of pancreatic cancer

Heather K. Lin, Dejah A. Blake, Tongrui Liu, Ruby Freeman, Gregory B. Lesinski, Lily Yang, and Sarwish Rafiq

SUPPLEMENTAL MATERIAL

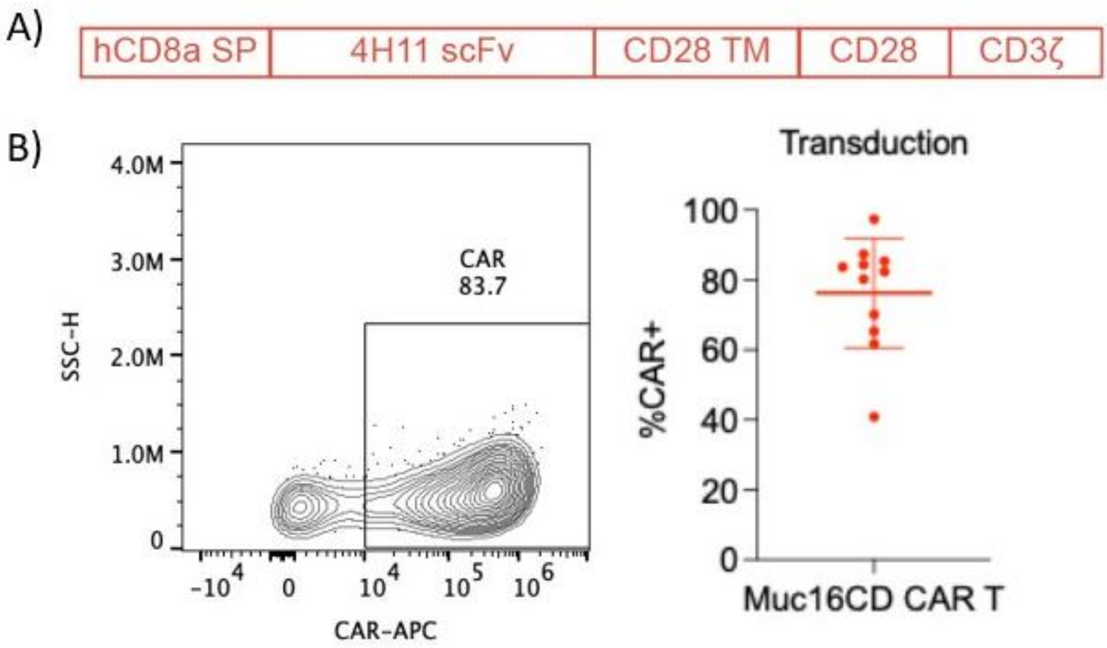

Figure S1. Muc16CD-directed CAR T cells are used in Figures 3 and 4. A) Second-generation CAR construct utilizing a human CD8a signal peptide (SP), 4H11 scFv binding domain, CD28 transmembrane domain, CD28 costimulatory domain, and CD3 zeta activation domain. B) Representative flow plot of CAR transduction (left) and quantification of transduction efficiencies of CAR T cells used in Figures 3 and 4 (right). Mean transduction efficiency was 76.2% with a standard deviation of 15.7%.

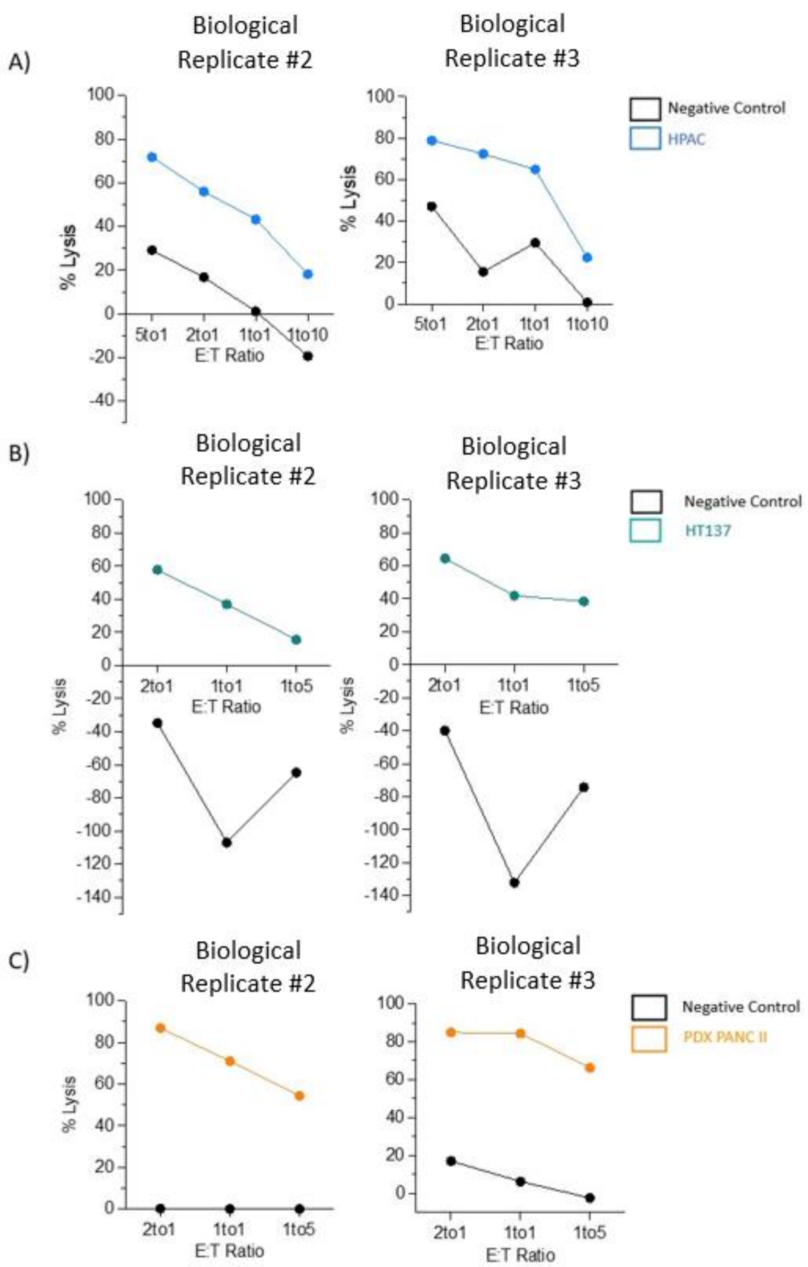

Figure S2. Replicates of cytotoxicity assays that are shown in Figure 3 of A) HPAC, B) HT137, and C) PANCII PDX cells. Negative values represent tumor growth compared to controls. CAR T cells derived from two additional healthy donor PBMCs as biological replicates. Negative control represents coculture with a Muc16CD negative cell line (either 3T3-GFPLuc or Panc1-GFPLuc cells).

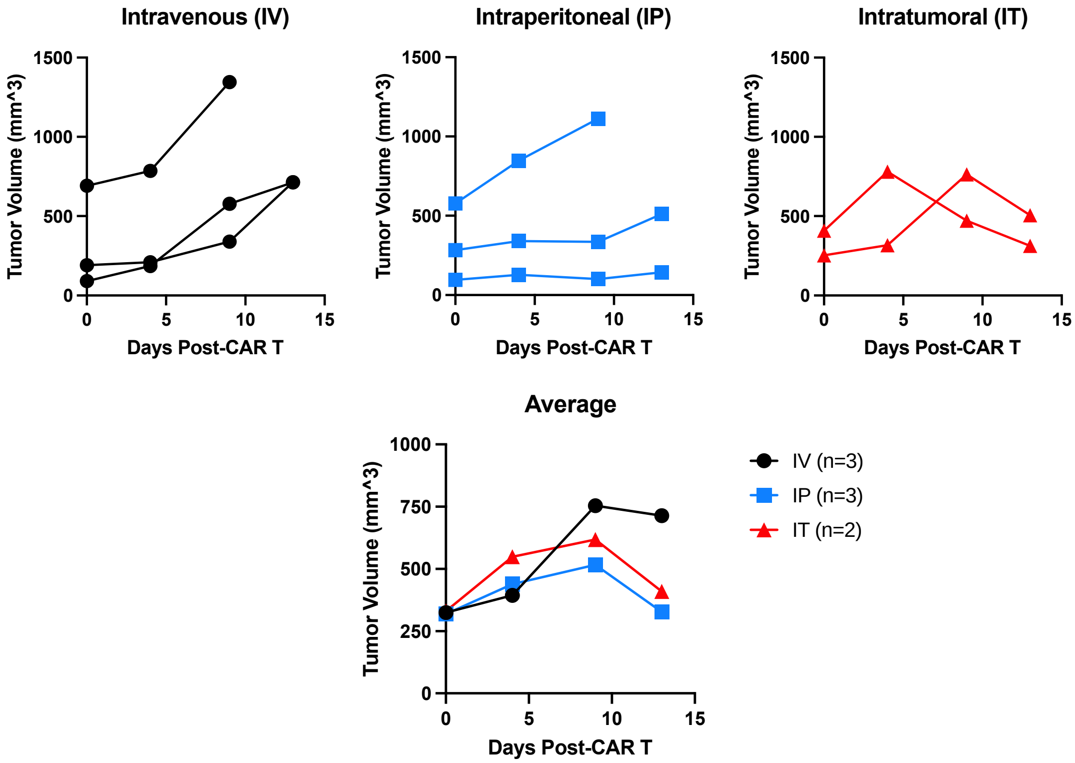

Figure S3. Intravenous and intraperitoneal administration of CAR T cells similarly affects tumor growth kinetics. Mice were engrafted with 1E6 Panc1-Muc16CD tumor cells and then treated with 2E6 CAR T cells by intravenous, intraperitoneal, or intratumoral administration.
