# Supplementary material for: Muc16CD is a novel CAR T cell target antigen for the treatment of pancreatic cancer
Source: Mol Ther Oncol. 2024 Sep 2;32(4):200868. doi: 10.1016/j.omton.2024.200868 (PMC11426034; doi:10.1016/j.omton.2024.200868)
Supplement: Document S2. Article plus supplemental information [file mmc2.pdf]

Molecular Therapy
Oncology
Original Article

Muc16CD is a novel CAR T cell target antigen for the treatment of pancreatic cancer

Heather K. Lin,[1] Dejah A. Blake,[1] Tongrui Liu,[2] Ruby Freeman,[1] Gregory B. Lesinski,[1,3] Lily Yang,[2,3] and Sarwish Rafiq[1,3]

[1]Department of Hematology and Medical Oncology, Emory University School of Medicine, Atlanta, GA, USA; [2]Department of Surgery, Emory University School of Medicine, Atlanta, GA, USA; [3]Winship Cancer Institute, Atlanta, GA, USA

Pancreatic cancer is an aggressive malignancy with a 5-year survival rate of 13% that remains refractory to current immunotherapies, such as chimeric antigen receptor (CAR) T cells. These engineered cells can produce robust anti-tumor responses but require a reliable tumor-associated antigen (TAA) target. Here, we describe the retained ectodomain of Muc16, Muc16CD, as a novel TAA for targeting by CAR T cell therapy in pancreatic cancer. We establish clinically relevant, endogenous Muc16 and Muc16CD expression in pancreatic tumor tissues for CAR T cell targeting. Muc16CD-directed CAR T cells can both recognize and activate in a polyfunctional manner in response to patient-derived pancreatic tumor cells. Last, we demonstrate that Muc16CD-directed CAR T cells can elicit an anti-tumor response *in vivo* with significantly enhanced tumor control and survival benefits in a pancreatic tumor model. Overall, these findings demonstrate the utility of Muc16CD-targeted CAR T cell therapy in the novel setting of pancreatic cancer.

INTRODUCTION

Pancreatic ductal adenocarcinoma (PDAC) is predicted to become the second leading cause of cancer-related deaths before 2030.[1] This heterogeneous malignancy is characterized by dense desmoplasia, extensive stromal cells and matrix, aberrant vasculature, and immunosuppressive cells, which contribute to poor immune cell infiltration.[2,3] Lack of effector T cells in the tumors leads to a poor response to various immunotherapies, including immune checkpoint inhibitors, in pancreatic cancer patients.[4,5] Providing tumor-specific T cells to overcome this lack of immune cell infiltration and dysfunction is a promising area of research. Chimeric antigen receptor (CAR) T cells redirect T cell specificity to tumor-associated antigens (TAAs). To date, results of clinical trials evaluating CAR T cell therapies in PDAC patients have shown limited efficacy. Those clinical trials used CAR T cells targeting epidermal growth factor receptor (EGFR), human epidermal growth factor receptor 2 (HER2), mesothelin, and Claudin 18.2.[6] Despite the limited efficacy of CAR T cells in these reports, the effectiveness, although limited, of T cell receptor-engineered T cells targeting oncogenic Kras in a case report suggests that engineered cellular therapies may have the potential to drive robust anti-tumor responses with the proper target and T cell activation.[7] Therefore, with an appropriate target, CAR T therapy has the potential to be an effective component of clinical therapy for PDAC.

Cancer antigen 125 (CA125) was discovered when the antibody OC125 was shown to react with antigens on ovarian cancer cells and in the patients' sera. The protein was then named Mucin 16 (Muc16) after the biochemical function was determined using this antibody.[8] Since then, it has been shown that Muc16 is an aberrant glycoprotein expressed by many solid tumors, such as ovarian,[9] breast,[10] lung,[11] and pancreatic cancer.[12] On the surface of normal epithelia, Muc16 protects the apical surface. However, increased expression of Muc16 on solid tumors contributes to tumor progression and metastasis while enabling immune escape by directly suppressing natural killer cells and macrophages.[8,13] Cleavage of the glycosylated portion of Muc16 reveals an oncogenic retained ectodomain (Muc16CD).[9] The first antibodies developed targeted the cleaved portion of Muc16, failing to detect the retained ectodomain on tumor cells.[8] Antibodies specific to the ectodomain, such as 4H11, which binds to the retained and non-glycosylated peptide backbone of Muc16, were next developed.[14] The 4H11 antibody was the basis of designing a single-chain variable fragment (scFv) binding domain for Muc16CD-targeting CAR T therapy.[15–17] In a phase 1 clinical trial, Muc16CD-specific CAR T cells were well tolerated and showed persistence in the blood in patients with recurrent ovarian cancer.[18,19] In this work, we utilize a second-generation CAR T cell with the same 4H11 scFv targeting the Muc16CD binding domain.

Since CA125 is elevated in patients with PDAC,[20] we aimed to investigate Muc16CD as a targetable TAA in pancreatic cancer. We demonstrate, for the first time, the anti-tumor activity of Muc16CD-targeting CAR T cells in pancreatic cancer. This work serves as proof of concept for the utility of Muc16CD-directed CAR T cells to treat pancreatic cancer.

RESULTS

High Muc16 expression confers worse survival in patients with pancreatic cancer

We first evaluated the clinical relevance of targeting Muc16 in resectable pancreatic cancer. To compare Muc16 expression in different

Received 5 June 2024; accepted 29 August 2024;
https://doi.org/10.1016/j.omton.2024.200868.

Correspondence: Sarwish Rafiq, Department of Hematology and Medical Oncology, Emory University School of Medicine, Atlanta, GA, USA.
E-mail: sarwish.rafiq@emory.edu

© 2024 The Author(s).
Published by Elsevier Inc. on behalf of The American Society of Gene and Cell Therapy.
This is an open access article under the CC BY license (http://creativecommons.org/licenses/by/4.0/).

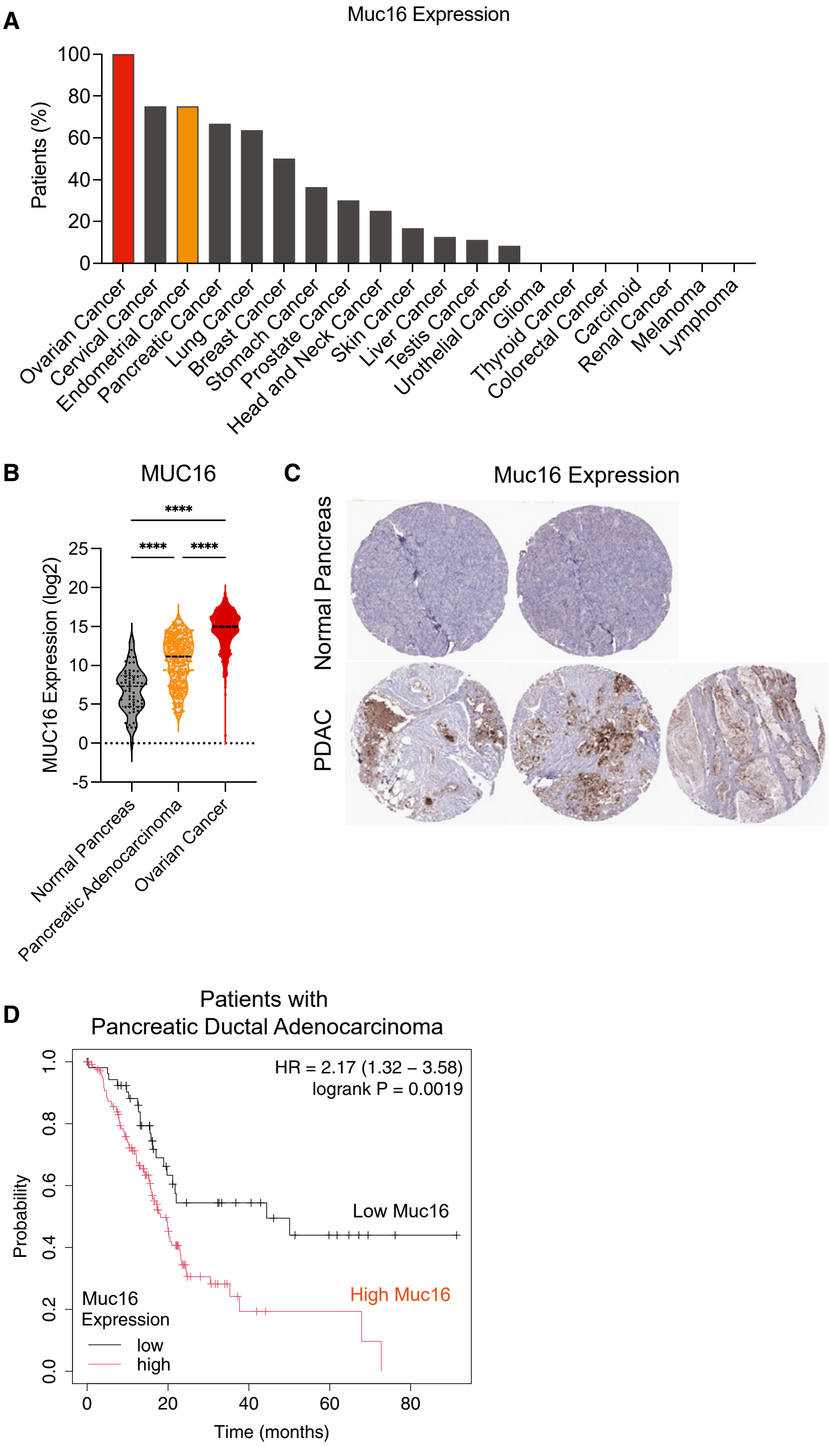

Figure 1. High Muc16 expression confers worse survival in patients with pancreatic cancer

(A) Percentage of patients with Muc16 expression in different cancer types as reported by the HPA. (B) Muc16 mRNA expression in normal pancreas, PDAC, and ovarian cancer tissue samples from TCGA Repository. Ovarian cancer is also plotted for reference. (C) Muc16 protein staining in normal pancreas and PDAC tissues. Image credit: HPA. (D) Overall survival of patients with PDAC stratified by high or low Muc16 expression via TCGA Repository. The plot was generated via KMPlotter. Data are represented as median with interquartile ranges in (B). The p values were determined by (B) two-way ANOVA and (D) log rank test. $^{*}p < 0.05$, $^{**}p < 0.01$, $^{***}p < 0.001$, $^{****}p < 0.0001$.

cancer types, we utilized the Human Protein Atlas (HPA).[21] Pancreatic cancer has the fourth-highest percentage of patients whose tumors express Muc16, following the well-characterized gynecological cancers, including ovarian, cervical, and endometrial (Figure 1A). Muc16 expression in PDAC was next analyzed using The Cancer Genome Atlas (TCGA) database. PDAC tumor tissues have significantly increased gene expression of MUC16 compared to normal pancreas tissue but decreased expression compared to ovarian cancer (Figure 1B, TCGA). This is further illustrated in normal pancreas and PDAC tissue samples (Figure 1C, HPA). When patients with PDAC are stratified by Muc16 expression levels, patients with high Muc16 expression have a significantly lower probability of survival than those with low Muc16 expression (Figure 1D, TCGA).

Muc16CD expression is upregulated in patient-derived pancreatic tumors

Muc16 is a known TAA that undergoes cleavage of the extracellular glycocalyx (CA-125), which is released into the blood. Serum CA-125 is used clinically as a biomarker of tumor burden, relapse, or response to treatment, primarily in the setting of ovarian cancer.[22] Cleavage of CA-125 reveals the retained ectodomain, Muc16CD, on the surface of the tumor cells, which can be targeted by CAR T cells (Figure 2A).[15,16] PDAC patient-derived xenograft (PDX) tumor samples were obtained during surgery from patients with resectable tumors to establish PDXs in mice (Table 1). To evaluate the targetable expression of Muc16CD in pancreatic cancer, PDAC PDX tumors were probed by immunofluorescence microscopy with a 4H11 antibody, which is the basis of the anti-Muc16CD CAR T cell scFv used in these studies. PDAC PDX tumors demonstrate a robust spectrum of Muc16CD expression (Figure 2B). To confirm the three-dimensional accessibility of Muc16CD expression for CAR T cell engagement, *in vivo* PANCII PDX tumors were harvested from mice and enzymatically digested to evaluate by flow cytometry. PANCII PDX tumor cells have robust surface expression of Muc16CD as compared to a control negative-expressing cell line, the KPC PDAC cell line (Figure 2C).

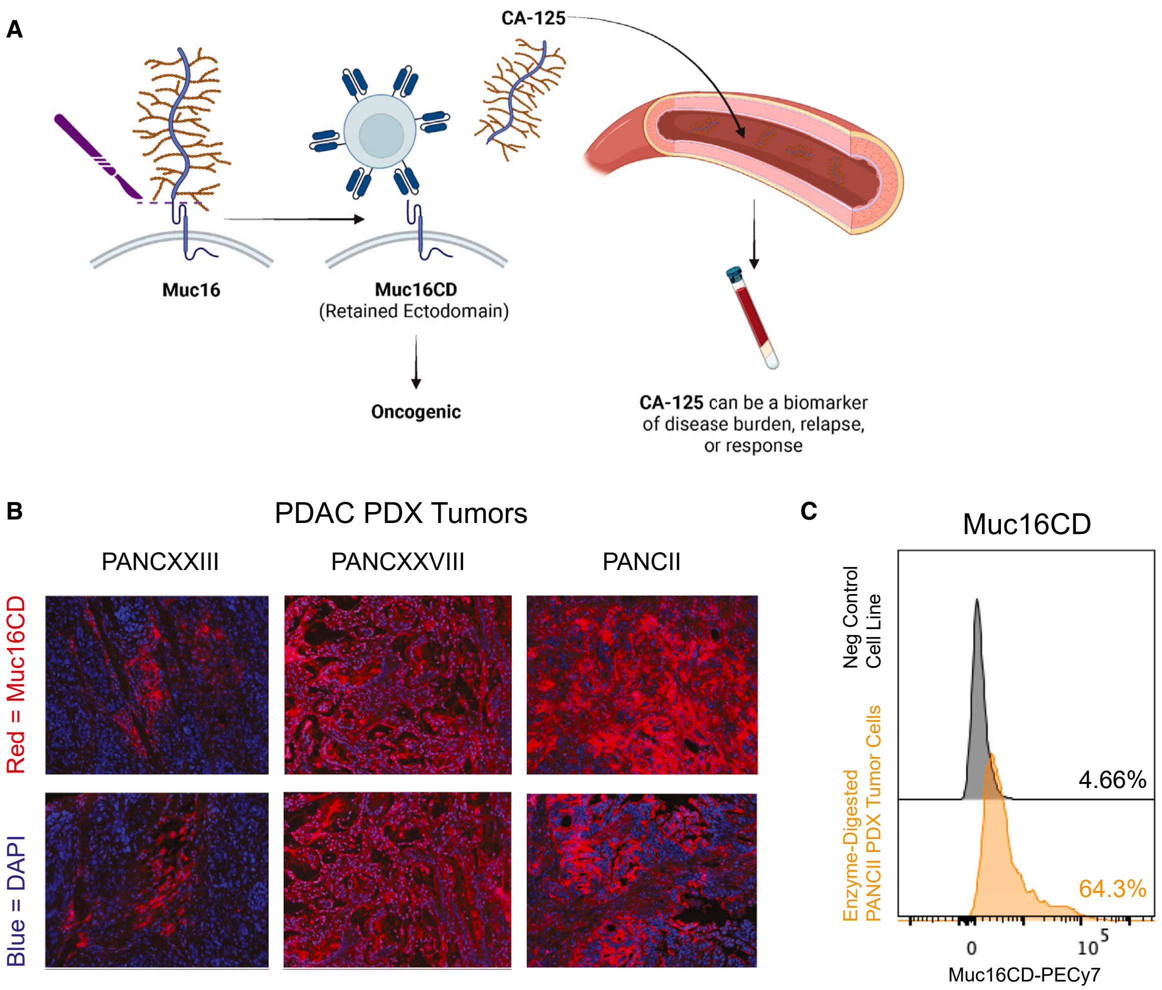

Figure 2. Muc16CD expression is upregulated in patient-derived pancreatic tumors

(A) Graphical representation of Muc16 cleavage to expose the retained ectodomain (Muc16CD) on the tumor cell surface. (B) Immunofluorescence microscopy images of PDAC PDX tumors (PANC XXIII, PANC II, and PANC XXVIII). Blue is DAPI nuclear staining, and red is expression of the retained ectodomain, Muc16CD. (C) A PANCII PDX *in vivo* tumor was freshly harvested from mice and enzymatically digested. Live cells were analyzed for Muc16CD surface expression by flow cytometry. The negative control cell line is the mouse KPC PDAC cell line that does not express Muc16CD.

CAR T cells recognize endogenous Muc16CD expression in pancreatic cancer cells

Next, we probed for endogenous surface Muc16CD expression on the human pancreatic cell lines: Panc1 and HPAC. We further explored Muc16CD expression in HT137,[23] a cancer-associated fibroblast (CAF) cell line, as well as PANCII PDX, a PDX-derived cell line. We found that HPAC, HT137, and PANCII PDX have detectable surface expression of endogenous Muc16CD compared to Panc1 (Figure 3A), which is known to have low Muc16 expression.[12] Notably, we observed lower expression in the *in vitro* PANCII PDX-derived cell line (17%; Figure 3A) than freshly enzyme-digested cells extracted from *in vivo* PANCII PDX tumors (64%; Figure 2C). To test whether endogenous Muc16CD is targetable by Muc6CD-directed CAR T cells, we evaluated antigen-specific cytotoxic function. Previously validated second-generation Muc16CD-directed CAR T cells with a 4H11 scFv binding domain and CD28 costimulatory domain[15–18,24] were generated with an average transduction efficiency of 76.2% (Figure S1). HPAC, HT137, and PANCII PDX tumor cells all demonstrate cytotoxicity in an effector-to-target dose-dependent manner (Figures 3B and S2). Of note, HPAC and HT137 were co-cultured with CAR T cells for 48 hours, with percent lysis determined by a flow-based killing assay, whereas PANCII PDX cells were co-cultured for 72 hours in an LDH-based killing assay (Figure 3B). To evaluate whether engagement of endogenous Muc16CD in an antigen-specific manner can induce an immune response in Muc16CD-directed CAR T cells, we cocultured CAR T cells with an antigen-irrelevant lymphoma cell line, Raji, or the antigen-relevant PANCII PDX cell line with endogenous Muc16CD expression. Coculture with PANCII PDX tumor cells leads CAR T cells to upregulate CD69 expression (Figure 3C), a marker of early T cell activation, and increases

Table 1. PDX tumor models

Patient ID	PANC XXIII	PANC XXVIII	PANCII
Type	pancreas	pancreas	pancreas
Gender	female	female	female
Race	African American	White	White
Age at surgery	67	63	75
Neoadjuvant chemotherapy	FOLFIRINOX + gemcitabine/paclitaxel	N/A	FOLFIRINOX
Recurrence/metastasis post surgery	yes	N/A	yes, liver
KRAS mutation	N/A	N/A	G12V

FOLFIRINOX, folinic acid, fluorouracil, irinotecan, and oxaliplatin.

expression of interleukin-2 (IL-2), interferon gamma (IFNg), tumor necrosis factor alpha (TNF-α), and granzyme B (GzmB), which are cytokines or effector molecules that contribute to activation, amplification, and persistence of CAR T cell function (Figure 3D).

Muc16CD-targeted CAR T cells improve survival in a pancreatic mouse model

Finally, to investigate the utility of Muc16CD-targeting CAR T cells to treat PDAC tumors *in vivo*, severe combined immunodeficiency (SCID)/beige mice were subcutaneously engrafted with 5E6 Panc1 tumor cells modified to express Muc16CD and GFP-luciferase (Figure 4A). Then, 7–10 days after tumor engraftment, mice were treated with a single intraperitoneal dose of either PBS or 5E6 Muc16CD-directed CAR T cells. Intraperitoneal administration of CAR T cells was not found to differ from intravenous administration in a separate experiment (Figure S3), which aligns with recent clinical trials.[19,25] To measure tumor burden, bioluminescence was measured on days 14, 28, 42, and 56. Mice were designated to reach the experimental endpoint when tumor volumes were greater than 2,000 mm^3 or ulcerated to measure survival. Muc16CD-directed CAR T cell-treated mice demonstrated significantly less pancreatic tumor burden compared to PBS-treated controls (Figures 4B and 4C). Muc16CD-directed CAR T cell treatment also significantly prolonged overall survival in tumor-bearing mice compared with PBS-treated controls (Figure 4D).

DISCUSSION

This work is the first to describe the expression and targetability of Muc16CD as a novel CAR T cell antigen TAA in pancreatic tumors. Clinically, Muc16 is upregulated in PDAC tumor tissues and confers poor survival in patients. Building upon this, we validated the endogenous expression of Muc16CD in a PDAC tumor cell line, a PDAC CAF cell line, and clinically relevant patient-derived tumors. We demonstrate that endogenous Muc16CD is targetable for cytotoxicity by Muc16CD-directed CAR T cells. The expression of endogenous Muc16CD on these pancreatic cell lines is also capable of activating Muc16CD-directed CAR T cells and invoking the expression of several cytokines required for a robust immune response. Finally, we show that administration of Muc16CD-directed CAR T cells controls pancreatic tumors *in vivo*, as evidenced by significantly improved tumor burden control and prolonged overall survival.

The upregulated expression of Muc16 on tumor cells contributes to both the metastasis and progression of disease in multiple malignancies, including PDAC,[8,26] which underscores the decreased survival observed in patients with high Muc16 gene expression (Figure 1D). One limitation of these data collected from TCGA is that it represents a patient population of resectable PDAC, which may not represent patients with other clinical stages of PDAC,[27] and therefore warrants further investigation. We anticipate that the well-characterized immunosuppressive microenvironment of PDAC likely abrogates the function of these infiltrating T cells.[28,29] Therefore, further studies of armored CAR T cells that deliver immunomodulatory molecules to engage the endogenous immune cells with CAR T cell therapy are of interest.[15,16,30,31]

In this work, we also provide the novel observation that Muc16CD is expressed endogenously on CAFs, which are a major component of the PDAC tumor microenvironment (TME). It is estimated that CAFs can comprise ∼80% of primary PDAC tumors and contribute to dense desmoplasia and reduced infiltration of immune cells.[32–34] Targeting of the CAF compartment has been explored with both mesothelin- and Claudin 18.2-targeted CAR T cells that require dual targeting of fibroblast-associated protein (FAP) with either secreted molecules or a mixed product with an anti-FAP CAR T cell.[35,36] Dual targeting of stromal and tumor cells has shown promise but relies on the careful coordination of both CAR targeting and secretion of FAPs. Here, we demonstrate the first antigen target for CAR T cells that has cytotoxic potential against both PDAC tumor and CAF cells. Like many other tumor biomarkers, the level of MUC16 expression is heterogeneous among different pancreatic PDX tumors and within the tumor cells in the same tumor. Although the level of MUC16 was high in tumor cells, we did find MUC16-positive fibroblast-like cells in tumor stroma areas. Further investigations will determine the types of stromal cells that are positive for MUC16 expression and the efficacy of Muc16CD-targeted CAR T cells in controlling the CAF compartment within the heterogeneous PDAC TME.

The unique structural features of Muc16 and mesothelin proteins represent critical biology that may contribute to the success or failure of existing CAR T therapies targeting these antigens in pancreatic cancer. Muc16 directly binds to mesothelin to facilitate cell-to-cell

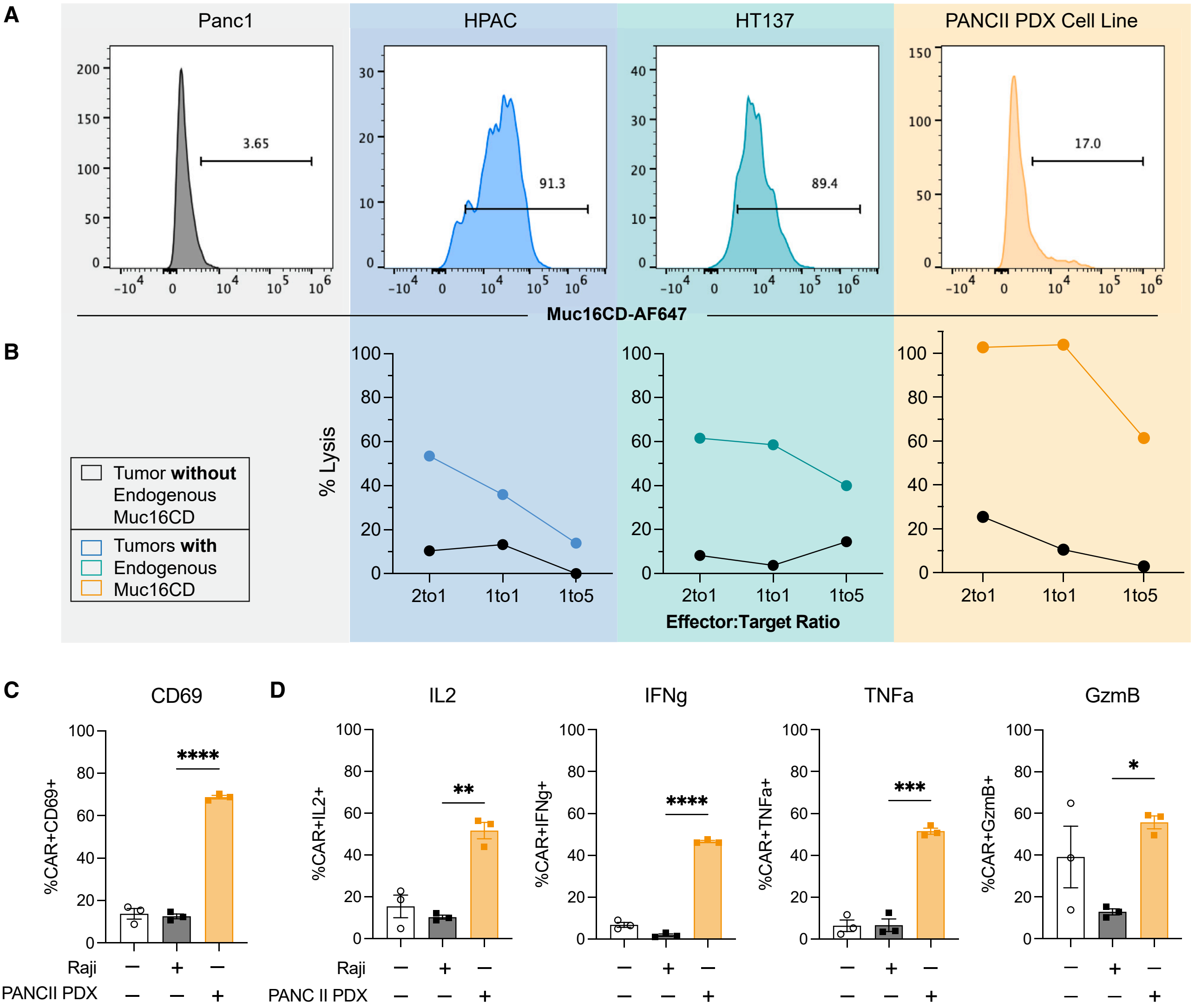

Figure 3. CAR T cells recognize endogenous Muc16CD expression in pancreatic tumor cells

(A) Endogenous Muc16CD expression on the tumor cell lines Panc1 (gray, negative control), HPAC (blue, human PDAC), HT137 (green, CAF), and PANCII PDX (orange, PDX-derived PDAC) were evaluated by flow cytometry. (B) Tumor cells were cocultured with CAR T cells at different E:T ratios. Cytotoxicity plots are representative of three independent experiments (see also Figure S1). HPAC and HT137 cells were co-cultured for 48 h, whereas PANCII PDX was co-cultured for 72 h. Negative control represents coculture with a Muc16CD-negative cell line (either 3T3-GFPLuc or Panc1-GFPLuc cells). Plots are representative of three independent experiments. (C and D) Muc16CD-directed CAR T cells upregulate (C) CD69 and (D) IL-2, IFNg, TNF-α, and GzmB when cocultured with PANCII PDX cells (cell line). Data represent mean with SEM in (C). The *p* values were determined by (C) two-way ANOVA. *$p < 0.05$, **$p < 0.01$, ***$p < 0.001$, ****$p < 0.0001$.

adhesion.[37] Like Muc16, mesothelin is a glycoprotein that undergoes cleavage to produce a soluble form released into the bloodstream.[38,39] Mesothelin-directed CAR T cells that are currently under clinical investigation target the released form of mesothelin.[39–41] However, clinical trials investigating mesothelin-directed CAR T cells to treat pancreatic cancer have had limited clinical efficacy thus far[42] or are currently ongoing (ClinicalTrials.gov: NCT03323944 and NCT04577326). This is likely due in part to the profoundly suppressive PDAC microenvironment limiting CAR T persistence. While mesothelin is robustly expressed in pancreatic cancers, one additional theory is that targeting the glycosylated portion is susceptible to cleavage, which could offer a mechanism that limits CAR T cell efficacy. This idea is supported by preclinical comparisons between membrane-proximal and -distal targeting domains in CAR T cells, showing that targeting membrane-proximal epitopes has greater anti-tumor efficacy than targeting distal epitopes.[43,44] In fact, targeting other membrane-proximal epitopes of Muc16 with a chimeric antibody (ch5E6) has shown promising preclinical results in the setting of pancreatic cancer.[45] However, these membrane-proximal epitopes for mesothelin have not yet entered clinical evaluation, nor

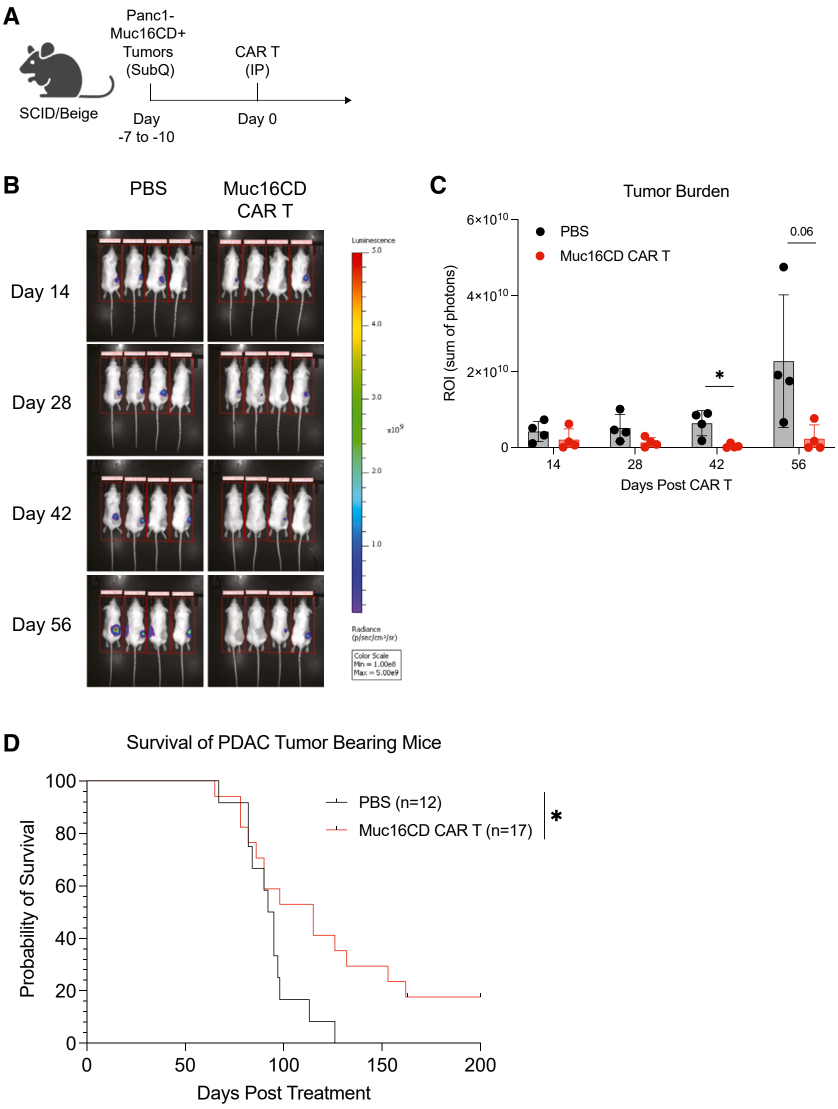

Figure 4. Muc16CD-targeted CAR T cells improve survival in a pancreatic mouse model

(A) Panc1-Muc16CD tumor cells (2.5–5E6) were subcutaneously engrafted into SCID/beige mice and subsequently treated intraperitoneally with Muc16CD-targeting CAR T cells (see also Figure S3) 5–10 days later. (B) Representative images of one independent experiment showing tumor burden measured by bioluminescence imaging at days 14, 28, 42, and 56. (C) Quantification of bioluminescence imaging. (D) Kaplan-Meier curve of OS (combination of three independent experiments). Data represent mean with standard deviation in (C). The p values were determined by (C) unpaired t tests and (D) log rank tests. *$p < 0.05$, **$p < 0.01$, ***$p < 0.001$, ****$p < 0.0001$.

is the safety of targeting them established in patients. Here, we highlight the advantage of targeting Muc16CD as a retained membrane-proximal epitope with a 4H11 antibody-based CAR T cell product that has undergone clinical evaluation in the setting of ovarian cancer and can be administered safely.[19] The establishment of Muc16CD as a novel tumor antigen in pancreatic cancers bolsters the development of CAR T therapies but also provides the opportunity to develop dual-targeting CAR T cells.

In conclusion, these findings demonstrate the utility and potential of targeting Muc16CD with CAR T therapy for the treatment of pancreatic cancer. Targeting Muc16CD as a dual targeting of tumor and CAF cells simplifies a multi-pronged approach to overcoming the PDAC TME. Simplifying the CAR target allows for further modifications and enhancements to CAR T cell engineering, such as targeting critical immune checkpoint pathways. Canonical immune checkpoint inhibitors such as PD-1, PD-L1, and CTLA4 have limited success in PDAC, highlighting an unmet need for incorporating novel immune checkpoint pathways into CAR T cell therapies, such as the Siglec-15[46] or vasointestinal peptide (VIP)/VIPR[47–49] pathways. Therefore, future studies of armoring Muc16CD-directed CAR T cells to target novel immune checkpoint pathways could additionally have powerful therapeutic utility.

MATERIALS AND METHODS

HPA and TCGA analysis

Data on expression of Muc16 in cancer types were acquired from Human Protein Atlas:CAB055127. Pancreas and pancreatic adenocarcinoma datasets were accessed via the NCI Genomic Data Commons (GDC) Data Portal: CPTAC-3, TCGA-PAAD. MUC16 expression counts were extracted, and log2 (unstranded counts) were calculated. Sample sheets associated with datasets were cross-referenced to identify "solid tissue normal" values designated as "normal pancreas" in Figure 1B. Muc16 staining of pancreatic tissues were obtained from the Human Protien Atlas: https://www.proteinatlas.org/ENSG00000181143-MUC16/pathology. KM Plotter – Pan-cancer RNA-Seq was utilized to stratify 177 patients with pancreatic ductal adenocarcinoma into "high" and "low" MUC16-expressing groups (Figure 1D).[50,51]

Healthy donor samples

Healthy donor peripheral blood mononuclear cells (PBMCs) were isolated from leukopaks (LifeSouth Blood Center, Dunwoody, GA, USA) with Ficoll-Paque Premium density gradient centrifugation (Cytiva, Marlborough, MA, USA).

Cell lines and reagents

Panc1 and HPAC cells were purchased from the ATCC (Manassas, VA, USA). HT137 cells were a gift from Dr. David Tuveson (Cold Spring Harbor Laboratory, NY, USA). PANC PDX tumors and cell

lines were established in L.Y.'s laboratory from surgically resected pancreatic cancer tissues following an approved institutional review board protocol (54023) at Emory University (Table 1). Retroviral packaging cells and tumor cell lines were maintained in DMEM. Human T cells were maintained in RPMI-1640 medium supplemented with 2 mM L-glutamine. All media were supplemented with 10% fetal bovine serum, 100 IU/mL penicillin, and 100 μg/mL streptomycin (Invitrogen, Waltham, MA, USA).

T cell isolation and modification

Human T cells were activated and transduced as described previously.[15,52] Briefly, PBMCs were activated with 4 μg/mL phytohemagglutinin (Remel, Lenexa, KS, USA) and supplemented with 100 IU/mL of IL-2 (R&D Systems, Minneapolis, MN, USA). Activated PBMCs were then transduced by centrifugation of filtered retroviral supernatant on RetroNectin-coated plates for 3 consecutive days (TakaraBio, Shiga, Japan). The Muc16CD-targeting CAR construct has a 4H11 scFv binding domain[14] and CD28 costimulatory domain, as described previously.[15–17] Retroviral packaging cells of the Muc16CD-targeting CAR construct (293-GalV9-4H11h28hz) were provided courtesy of Dr. Renier Brentjens (Roswell Park Cancer Institute, Buffalo, NY, USA).

Cytotoxicity assays

HPAC-GFPLuc and HT137-RFP tumor cells were co-cultured with CAR T cells at defined effector-to-target (E:T) ratios for 48 h. Cell death was determined by flow cytometry with 123eCount beads (Thermo Fisher Scientific, Waltham MA) to define the number of live GFP+ cells. Percent lysis was calculated by (1 − [experimental well/tumor alone well]) × 100.

The PANCII PDX cell line and CAR T cells were co-cultured at defined E:T ratios. Cell death was determined by CyQUANT LDH Cytotoxicity Assay (Invitrogen) at 72 h. Percent lysis was calculated by (experimental well)/(complete lysis well) per the manufacturer's protocol.

CAR T cell activation cocultures

Muc16CD-directed CAR T cells were cocultured with the Raji-GFPLuc or PANCII PDX cell line for 24 h at a 1:1 E:T ratios. Cells were stained for surface CD69, TNF-α, granzyme B (BioLegend, San Diego, CA, USA), IL-2, and IFNg (Invitrogen) expression. For cytokine markers, cells were incubated with GolgiStop (BD Biosciences, Franklin Lakes, NJ, USA) for 4 h before intracellular staining.

Flow cytometry

Flow cytometry was used to determine the transduction efficiency of transduced cells following staining with fluorophore-conjugated idiotype antibodies (clone 226G) that detect the Muc16CD-targeted CAR (clone 4H11) generated at the Memorial Sloan Kettering Cancer Center Antibody and Bioresource Core Facility (New York, NY, USA). To detect Muc16CD expression on tumor cells, an anti-Muc16CD antibody (clone 4H11) was generated at the Memorial Sloan Kettering Cancer Center Antibody and Bioresource Core Facility and conjugated with APC (Abcam, Cambridge, UK). Surface staining was performed for CD69 (clone FN50, BioLegend). Intracellular staining was performed with a Cytofix/Cytoperm Kit according to the manufacturer's protocol (BD Biosciences) and stained for IL-2 (clone MQ1-7H12, Invitrogen), IFNg (clone 4S.B3, Invitrogen), TNF-α (clone MAb11, BioLegend), and GzmB (clone 7F11A10, BioLegend).

Immunofluorescence microscopy

To prepare tumor slices for *ex vivo* imaging experiments, entire tumors were collected and immersed into optimal cutting temperature compound, followed by being snap frozen in liquid nitrogen. Frozen tumor sections (8 μm) of tumor tissues were blocked with goat serum and mouse immunoglobulin G and then stained with a mouse anti-Muc16CD monoclonal antibody (clone 4H11, Memorial Sloan Kettering Antibody Core), followed by a goat anti-mouse 555 dye-labeled secondary antibody. Hoechst 33342, trihydrochloride, trihydrate (DAPI) substrate solution was used to detect nucleic acid stains. Fluorescence images were taken using an inverted fluorescence microscope (BZ-X710 All-in-One, Keyence).

Xenograft PDAC mouse experiments

All experiments were performed in compliance with the relevant ethics regulations in accordance with the Institutional Animal Care and Use Committee-approved protocols (PROTO202000015 and PROTO201700779). For tumor volume of Panc1-bearing mice, CB17.Cg-PrkdcscidLystbg-J/Crl (SCID/beige, Charles River Laboratories, Wilmington, MA, USA) mice were engrafted subcutaneously with Panc1-Muc16CD-GFPLuc+ tumor cells and then treated intraperitoneally with CAR T cells 7–10 days later. Calipers were used to measure tumor volume. The endpoint was defined as tumors greater than 2,000 mm^3 or ulceration.

Statistics and software

The Kaplan-Meier method was used to generate overall survival (OS) probabilities, and survival curves were compared between the groups using the log rank test. Paired sample t test, independent sample t test, or two-way ANOVA were employed as appropriate. An alpha of 0.05 was used. All statistical analyses were conducted using GraphPad Prism. Flow cytometry data were analyzed with FlowJo. Visualization of data or illustrations was done in Adobe Illustrator or BioRender.

DATA AND CODE AVAILABILITY

The data that support the findings of this study are available from the corresponding author upon request.

ACKNOWLEDGMENTS

Research reported in this publication was supported by Winship Shared Resources (P30CA138292), specifically the Pediatrics/Winship Flow Cytometry Core. The Division of Animal Resources supported animal studies. The authors would like to thank Dr. Renier Brentjens for contributing the Muc16CD-directed CAR constructs and the Memorial Sloan Kettering Cancer Center Antibody and Bioresource Core Facility for Muc16CD-directed and CAR-directed antibodies. Research reported in this publication was supported in part by the Donaldson Charitable Trust Research Synergy Fund Award (to S.R.), a Georgia Center for Oncology Research and Education Pilot Grant (to S.R.), a Winship Cancer Institute ACS Pilot Grant (to S.R.), the Winship Invest$ Pilot Project Program (to S.R.), the Mary Kay Ash Foundation (to S.R.), the Emory University

Research Committee (to S.R.), the Georgia CTSA's Pilot Grants Program (to S.R.), the ASH Minority Hematology Graduate Award (to D.B.), and NIHR41CA247165 (to L.Y.).

AUTHOR CONTRIBUTIONS

H.K.L., D.A.B., and S.R. conceived, designed, and developed the experimental study methodology. H.K.L., D.A.B., T.L., and R.F. performed experiments and acquired data. H.K.L., D.A.B., T.L., R.F., G.B.L., L.Y., and SR discussed results and interpreted data. H.K.L., D.A.B., and S.R. wrote and revised the manuscript. G.B.L. and L.Y. further revised the manuscript. All authors contributed to the final manuscript.

DECLARATION OF INTERESTS

G.B.L. has received research funding through a sponsored research agreement between Emory University and Merck and Co., Bristol-Myers Squibb, Boehringer Ingelheim, and Vaccinex. L.Y. is president of MIGRA-Therapeutics, LLC. S.R. is on the scientific advisory board for Celyad Oncology.

SUPPLEMENTAL INFORMATION

Supplemental information can be found online at https://doi.org/10.1016/j.omton.2024.200868.

REFERENCES

1. (2022). Cancer Facts & Figures 2022 (American Cancer Society).
2. Marofi, F., Motavalli, R., Safonov, V.A., Thangavelu, L., Yumashev, A.V., Alexander, M., Shomali, N., Chartrand, M.S., Pathak, Y., Jarahian, M., et al. (2021). CAR T cells in solid tumors: challenges and opportunities. Stem Cell Res. Ther. *12*, 81. https://doi.org/10.1186/s13287-020-02128-1.
3. Carstens, J.L., Correa de Sampaio, P., Yang, D., Barua, S., Wang, H., Rao, A., Allison, J.P., LeBleu, V.S., and Kalluri, R. (2017). Spatial computation of intratumoral T cells correlates with survival of patients with pancreatic cancer. Nat. Commun. *8*, 15095. https://doi.org/10.1038/ncomms15095.
4. Principe, D.R., Korc, M., Kamath, S.D., Munshi, H.G., and Rana, A. (2021). Trials and tribulations of pancreatic cancer immunotherapy. Cancer Lett. *504*, 1–14. https://doi.org/10.1016/j.canlet.2021.01.031.
5. Weiss, G.J., Blaydorn, L., Beck, J., Bornemann-Kolatzki, K., Urnovitz, H., Schütz, E., and Khemka, V. (2018). Phase Ib/II study of gemcitabine, nab-paclitaxel, and pembrolizumab in metastatic pancreatic adenocarcinoma. Invest. N. Drugs *36*, 96–102. https://doi.org/10.1007/s10637-017-0525-1.
6. Yeo, D., Giardina, C., Saxena, P., and Rasko, J.E.J. (2022). The next wave of cellular immunotherapies in pancreatic cancer. Mol. Ther. Oncolytics *24*, 561–576. https://doi.org/10.1016/j.omto.2022.01.010.
7. Leidner, R., Sanjuan Silva, N., Huang, H., Sprott, D., Zheng, C., Shih, Y.-P., Leung, A., Payne, R., Sutcliffe, K., Cramer, J., et al. (2022). Neoantigen T-Cell Receptor Gene Therapy in Pancreatic Cancer. N. Engl. J. Med. Overseas. Ed. *386*, 2112–2119. https://doi.org/10.1056/NEJMoa2119662.
8. Aithal, A., Rauth, S., Kshirsagar, P., Shah, A., Lakshmanan, I., Junker, W.M., Jain, M., Ponnusamy, M.P., and Batra, S.K. (2018). MUC16 as a novel target for cancer therapy. Expert Opin. Ther. Targets *22*, 675–686. https://doi.org/10.1080/14728222.2018.1498845.
9. Rao, T.D., Tian, H., Ma, X., Yan, X., Thapi, S., Schultz, N., Rosales, N., Monette, S., Wang, A., Hyman, D.M., et al. (2015). Expression of the Carboxy-Terminal Portion of MUC16/CA125 Induces Transformation and Tumor Invasion. PLoS One *10*, e0126633. https://doi.org/10.1371/journal.pone.0126633.
10. Lakshmanan, I., Ponnusamy, M.P., Das, S., Chakraborty, S., Haridas, D., Mukhopadhyay, P., Lele, S.M., and Batra, S.K. (2012). MUC16 induced rapid G2/M transition via interactions with JAK2 for increased proliferation and anti-apoptosis in breast cancer cells. Oncogene *31*, 805–817. https://doi.org/10.1038/onc.2011.297.
11. Lakshmanan, I., Salfity, S., Seshacharyulu, P., Rachagani, S., Thomas, A., Das, S., Majhi, P.D., Nimmakayala, R.K., Vengoji, R., Lele, S.M., et al. (2017). MUC16 Regulates TSPYL5 for Lung Cancer Cell Growth and Chemoresistance by Suppressing p53. Clin. Cancer Res. *23*, 3906–3917. https://doi.org/10.1158/1078-0432.Ccr-16-2530.
12. Haridas, D., Chakraborty, S., Ponnusamy, M.P., Lakshmanan, I., Rachagani, S., Cruz, E., Kumar, S., Das, S., Lele, S.M., Anderson, J.M., et al. (2011). Pathobiological implications of MUC16 expression in pancreatic cancer. PLoS One *6*, e26839. https://doi.org/10.1371/journal.pone.0026839.
13. Lee, K., Perry, K., Xu, M., Veillard, I., Kumar, R., Rao, T.D., Rueda, B.R., Spriggs, D.R., and Yeku, O.O. (2024). Structural basis for antibody recognition of the proximal MUC16 ectodomain. J. Ovarian Res. *17*, 41. https://doi.org/10.1186/s13048-024-01373-9.
14. Dharma Rao, T., Park, K.J., Smith-Jones, P., Iasonos, A., Linkov, I., Soslow, R.A., and Spriggs, D.R. (2010). Novel monoclonal antibodies against the proximal (carboxy-terminal) portions of MUC16. Appl. Immunohistochem. Mol. Morphol. *18*, 462–472. https://doi.org/10.1097/PAI.0b013e3181dbfcd2.
15. Rafiq, S., Yeku, O.O., Jackson, H.J., Purdon, T.J., van Leeuwen, D.G., Drakes, D.J., Song, M., Miele, M.M., Li, Z., Wang, P., et al. (2018). Targeted delivery of a PD-1-blocking scFv by CAR-T cells enhances anti-tumor efficacy in vivo. Nat. Biotechnol. *36*, 847–856. https://doi.org/10.1038/nbt.4195.
16. Yeku, O.O., Purdon, T.J., Koneru, M., Spriggs, D., and Brentjens, R.J. (2017). Armored CAR T cells enhance antitumor efficacy and overcome the tumor microenvironment. Sci. Rep. *7*, 10541. https://doi.org/10.1038/s41598-017-10940-8.
17. Koneru, M., Purdon, T.J., Spriggs, D., Koneru, S., and Brentjens, R.J. (2015). IL-12 secreting tumor-targeted chimeric antigen receptor T cells eradicate ovarian tumors *in vivo*. OncoImmunology *4*, e994446. https://doi.org/10.4161/2162402X.2014.994446.
18. Koneru, M., O'Cearbhaill, R., Pendharkar, S., Spriggs, D.R., and Brentjens, R.J. (2015). A phase I clinical trial of adoptive T cell therapy using IL-12 secreting MUC-16(ecto) directed chimeric antigen receptors for recurrent ovarian cancer. J. Transl. Med. *13*, 102. https://doi.org/10.1186/s12967-015-0460-x.
19. O'Cearbhaill, R.E., Park, J.H., Halton, E.F., Diamonte, C.R., Mead, E., Lakhman, Y., Kane, P., Riviere, I.C., and Brentjens, R.J. (2020). A phase I clinical trial of autologous chimeric antigen receptor (CAR) T cells genetically engineered to secrete IL-12 and to target the MUC16ecto antigen in patients (pts) with MUC16ecto+ recurrent high-grade serous ovarian cancer (HGSOC). Gynecol. Oncol. *159*, 42. https://doi.org/10.1016/j.ygyno.2020.06.089.
20. Napoli, N., Kauffmann, E.F., Ginesini, M., Lami, L., Lombardo, C., Vistoli, F., Campani, D., and Boggi, U. (2023). Ca 125 is an independent prognostic marker in resected pancreatic cancer of the head of the pancreas. Updates Surg. *75*, 1481–1496. https://doi.org/10.1007/s13304-023-01587-4.
21. Uhlen, M., Zhang, C., Lee, S., Sjöstedt, E., Fagerberg, L., Bidkhori, G., Benfeitas, R., Arif, M., Liu, Z., Edfors, F., et al. (2017). A pathology atlas of the human cancer transcriptome. Science *357*, eaan2507. https://doi.org/10.1126/science.aan2507.
22. Charkhchi, P., Cybulski, C., Gronwald, J., Wong, F.O., Narod, S.A., and Akbari, M.R. (2020). CA125 and Ovarian Cancer: A Comprehensive Review. Cancers *12*, 3730. https://doi.org/10.3390/cancers12123730.
23. Elyada, E., Bolisetty, M., Laise, P., Flynn, W.F., Courtois, E.T., Burkhart, R.A., Teinor, J.A., Belleau, P., Biffi, G., Lucito, M.S., et al. (2019). Cross-Species Single-Cell Analysis of Pancreatic Ductal Adenocarcinoma Reveals Antigen-Presenting Cancer-Associated Fibroblasts. Cancer Discov. *9*, 1102–1123. https://doi.org/10.1158/2159-8290.Cd-19-0094.
24. Chekmasova, A.A., Rao, T.D., Nikhamin, Y., Park, K.J., Levine, D.A., Spriggs, D.R., and Brentjens, R.J. (2010). Successful eradication of established peritoneal ovarian tumors in SCID-Beige mice following adoptive transfer of T cells genetically targeted to the MUC16 antigen. Clin. Cancer Res. *16*, 3594–3606. https://doi.org/10.1158/1078-0432.CCR-10-0192.
25. Zhang, H., Yang, Z., Zhu, X., Li, J., Gao, Y., Zhang, Y., Tong, Z., Fu, Q., Bao, X., Li, B., et al. (2024). Phase I trial of hypoxia-responsive CEA CAR-T cell therapy in patients with heavily pretreated solid tumor via intraperitoneal or intravenous transfusion. J. Clin. Oncol. *42*, 3514. https://doi.org/10.1200/JCO.2024.42.16_suppl.3514.
26. Thomas, D., Sagar, S., Liu, X., Lee, H.-R., Grunkemeyer, J.A., Grandgenett, P.M., Caffrey, T., O'Connell, K.A., Swanson, B., Marcos-Silva, L., et al. (2021). Isoforms of MUC16 activate oncogenic signaling through EGF receptors to enhance the progression of pancreatic cancer. Mol. Ther. *29*, 1557–1571. https://doi.org/10.1016/j.ymthe.2020.12.029.

27. Peran, I., Madhavan, S., Byers, S.W., and McCoy, M.D. (2018). Curation of the Pancreatic Ductal Adenocarcinoma Subset of the Cancer Genome Atlas Is Essential for Accurate Conclusions about Survival-Related Molecular Mechanisms. Clin. Cancer Res. *24*, 3813–3819. https://doi.org/10.1158/1078-0432.Ccr-18-0290.

28. Falcomatà, C., Bärthel, S., Schneider, G., Rad, R., Schmidt-Supprian, M., and Saur, D. (2023). Context-Specific Determinants of the Immunosuppressive Tumor Microenvironment in Pancreatic Cancer. Cancer Discov. *13*, 278–297. https://doi.org/10.1158/2159-8290.Cd-22-0876.

29. Ho, W.J., Jaffee, E.M., and Zheng, L. (2020). The tumour microenvironment in pancreatic cancer - clinical challenges and opportunities. Nat. Rev. Clin. Oncol. *17*, 527–540. https://doi.org/10.1038/s41571-020-0363-5.

30. Rafiq, S., Hackett, C.S., and Brentjens, R.J. (2020). Engineering strategies to overcome the current roadblocks in CAR T cell therapy. Nat. Rev. Clin. Oncol. *17*, 147–167. https://doi.org/10.1038/s41571-019-0297-y.

31. Evans, A.N., Lin, H.K., Hossian, A.K.M.N., and Rafiq, S. (2021). Using Adoptive Cellular Therapy for Localized Protein Secretion. Cancer J. *27*, 159–167. https://doi.org/10.1097/ppo.0000000000000510.

32. Stouten, I., van Montfoort, N., and Hawinkels, L.J.A.C. (2023). The Tango between Cancer-Associated Fibroblasts (CAFs) and Immune Cells in Affecting Immunotherapy Efficacy in Pancreatic Cancer. Int. J. Mol. Sci. *24*, 8707. https://doi.org/10.3390/ijms24108707.

33. Ware, M.B., El-Rayes, B.F., and Lesinski, G.B. (2020). Mirage or long-awaited oasis: reinvigorating T-cell responses in pancreatic cancer. J. Immunother. Cancer *8*, e001100. https://doi.org/10.1136/jitc-2020-001100.

34. Horvat, N.K., Karpovsky, I., Phillips, M., Wyatt, M.M., Hall, M.A., Herting, C.J., Hammons, J., Mahdi, Z., Moffitt, R.A., Paulos, C.M., and Lesinski, G.B. (2024). Clinically relevant orthotopic pancreatic cancer models for adoptive T cell transfer therapy. J. Immunother. Cancer *12*, e008086. https://doi.org/10.1136/jitc-2023-008086.

35. Wehrli, M., Guinn, S., Birocchi, F., Kuo, A., Sun, Y., Larson, R.C., Almazan, A.J., Scarfò, I., Bouffard, A.A., Bailey, S.R., et al. (2024). Mesothelin CAR T Cells Secreting Anti-FAP/Anti-CD3 Molecules Efficiently Target Pancreatic Adenocarcinoma and its Stroma. Clin. Cancer Res. *30*, 1859–1877. https://doi.org/10.1158/1078-0432.Ccr-23-3841.

36. Liu, Y., Sun, Y., Wang, P., Li, S., Dong, Y., Zhou, M., Shi, B., Jiang, H., Sun, R., and Li, Z. (2023). FAP-targeted CAR-T suppresses MDSCs recruitment to improve the antitumor efficacy of claudin18.2-targeted CAR-T against pancreatic cancer. J. Transl. Med. *21*, 255. https://doi.org/10.1186/s12967-023-04080-z.

37. Chen, S.-H., Hung, W.-C., Wang, P., Paul, C., and Konstantopoulos, K. (2013). Mesothelin Binding to CA125/MUC16 Promotes Pancreatic Cancer Cell Motility and Invasion via MMP-7 Activation. Sci. Rep. *3*, 1870. https://doi.org/10.1038/srep01870.

38. Montemagno, C., Cassim, S., Pouyssegur, J., Broisat, A., and Pagès, G. (2020). From Malignant Progression to Therapeutic Targeting: Current Insights of Mesothelin in Pancreatic Ductal Adenocarcinoma. Int. J. Mol. Sci. *21*, 4067. https://doi.org/10.3390/ijms21114067.

39. Zhai, X., Mao, L., Wu, M., Liu, J., and Yu, S. (2023). Challenges of Anti-Mesothelin CAR-T-Cell Therapy. Cancers *15*, 1357. https://doi.org/10.3390/cancers15051357.

40. Morello, A., Sadelain, M., and Adusumilli, P.S. (2016). Mesothelin-Targeted CARs: Driving T Cells to Solid Tumors. Cancer Discov. *6*, 133–146. https://doi.org/10.1158/2159-8290.CD-15-0583.

41. Luckett, T., Abudula, M., Ireland, L., Glenn, M., Bellomo, G., Stafferton, R., Halloran, C., Ghaneh, P., Jones, R., Schmid, M.C., and Mielgo, A. (2024). Mesothelin Secretion by Pancreatic Cancer Cells Co-opts Macrophages and Promotes Metastasis. Cancer Res. *84*, 527–544. https://doi.org/10.1158/0008-5472.Can-23-1542.

42. Beatty, G.L., O'Hara, M.H., Lacey, S.F., Torigian, D.A., Nazimuddin, F., Chen, F., Kulikovskaya, I.M., Soulen, M.C., McGarvey, M., Nelson, A.M., et al. (2018). Activity of Mesothelin-Specific Chimeric Antigen Receptor T Cells Against Pancreatic Carcinoma Metastases in a Phase 1 Trial. Gastroenterology *155*, 29–32. https://doi.org/10.1053/j.gastro.2018.03.029.

43. Tomar, S., Zhang, J., Khanal, M., Hong, J., Venugopalan, A., Jiang, Q., Sengupta, M., Miettinen, M., Li, N., Pastan, I., et al. (2022). Development of Highly Effective Anti-Mesothelin hYP218 Chimeric Antigen Receptor T Cells With Increased Tumor Infiltration and Persistence for Treating Solid Tumors. Mol. Cancer Therapeut. *21*, 1195–1206. https://doi.org/10.1158/1535-7163.Mct-22-0073.

44. Zhang, Z., Jiang, D., Yang, H., He, Z., Liu, X., Qin, W., Li, L., Wang, C., Li, Y., Li, H., et al. (2019). Modified CAR T cells targeting membrane-proximal epitope of mesothelin enhances the antitumor function against large solid tumor. Cell Death Dis. *10*, 476. https://doi.org/10.1038/s41419-019-1711-1.

45. Shah, A., Chaudhary, S., Lakshmanan, I., Aithal, A., Kisling, S.G., Sorrell, C., Marimuthu, S., Gautam, S.K., Rauth, S., Kshirsagar, P., et al. (2023). Chimeric antibody targeting unique epitope on onco-mucin16 reduces tumor burden in pancreatic and lung malignancies. npj Precis. Oncol. *7*, 74. https://doi.org/10.1038/s41698-023-00423-7.

46. Chen, X., Mo, S., Zhang, Y., Ma, H., Lu, Z., Yu, S., and Chen, J. (2022). Analysis of a novel immune checkpoint, Siglec-15, in pancreatic ductal adenocarcinoma. J. Pathol. Clin. Res. *8*, 268–278. https://doi.org/10.1002/cjp2.260.

47. Ravindranathan, S., Passang, T., Li, J.-M., Wang, S., Dhamsania, R., Ware, M.B., Zaidi, M.Y., Zhu, J., Cardenas, M., Liu, Y., et al. (2022). Targeting vasoactive intestinal peptide-mediated signaling enhances response to immune checkpoint therapy in pancreatic ductal adenocarcinoma. Nat. Commun. *13*, 6418. https://doi.org/10.1038/s41467-022-34242-4.

48. Lin, H., Freeman, R., Evans, A., Fnu, T.P., Liu, T., Christensen, E., Fei, R., Sinha, T., Ravindranathan, S., Yang, L., et al. (2022). 310 Engineering optimal CAR T cells to overcome pancreatic tumors with secreted antagonistic peptides. J. Immunother. Cancer *10*, A325–A326. https://doi.org/10.1136/jitc-2022-SITC2022.0310.

49. Lin, H., Blake, D., Mudigonda, A., Evans, A.N., Freeman, R., Sinha, T., Fei, F., Christensen, E., Fnu, T.P., Ravindranathan, S., et al. (2023). Antagonizing Vasoactive Intestinal Peptide (VIP) Receptors on CAR T Cells Improves Efficacy and Persistence. Blood *142*, 4806. https://doi.org/10.1182/blood-2023-182952.

50. Gyorffy, B. (2024). Integrated analysis of public datasets for the discovery and validation of survival-associated genes in solid tumors. Innovation (Camb) *5*, 100625. https://doi.org/10.1016/j.xinn.2024.100625.

51. Gyorffy, B. (2024). Transcriptome-level discovery of survival-associated biomarkers and therapy targets in non-small-cell lung cancer. Br. J. Pharmacol. *181*, 362–374. https://doi.org/10.1111/bph.16257.

52. Rafiq, S., Purdon, T.J., Daniyan, A.F., Koneru, M., Dao, T., Liu, C., Scheinberg, D.A., and Brentjens, R.J. (2017). Optimized T-cell receptor-mimic chimeric antigen receptor T cells directed toward the intracellular Wilms Tumor 1 antigen. Leukemia *31*, 1788–1797. https://doi.org/10.1038/leu.2016.373.

OMTON, Volume *32*

Supplemental information

Muc16CD is a novel CAR T cell target antigen for the treatment of pancreatic cancer

Heather K. Lin, Dejah A. Blake, Tongrui Liu, Ruby Freeman, Gregory B. Lesinski, Lily Yang, and Sarwish Rafiq

SUPPLEMENTAL MATERIAL

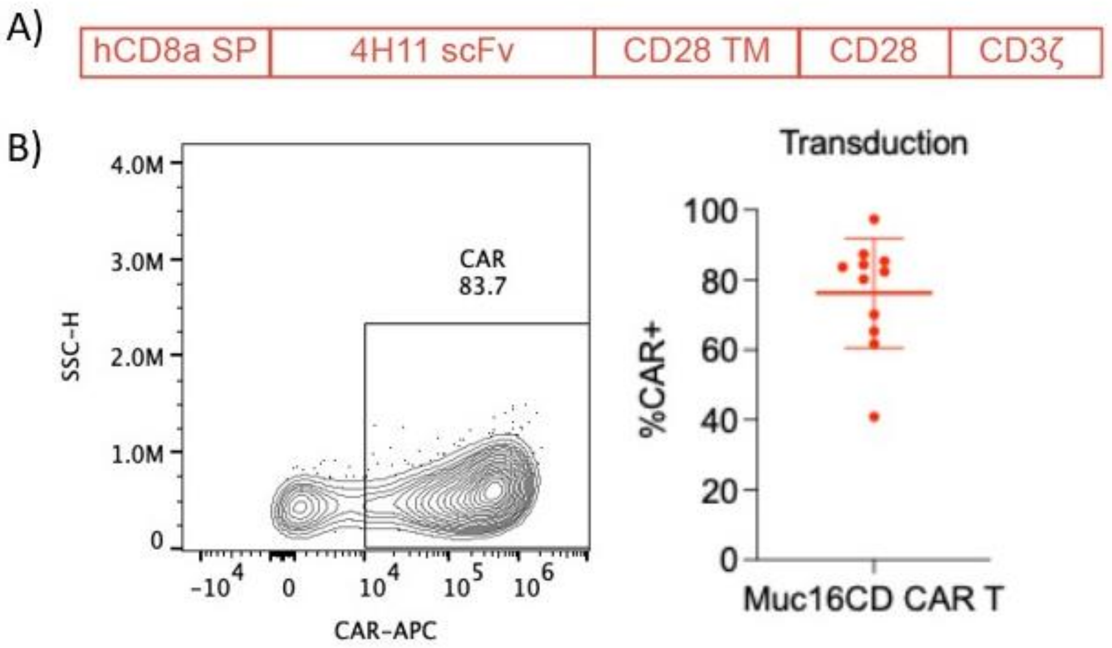

Figure S1. Muc16CD-directed CAR T cells are used in Figures 3 and 4. A) Second-generation CAR construct utilizing a human CD8a signal peptide (SP), 4H11 scFv binding domain, CD28 transmembrane domain, CD28 costimulatory domain, and CD3 zeta activation domain. B) Representative flow plot of CAR transduction (left) and quantification of transduction efficiencies of CAR T cells used in Figures 3 and 4 (right). Mean transduction efficiency was 76.2% with a standard deviation of 15.7%.

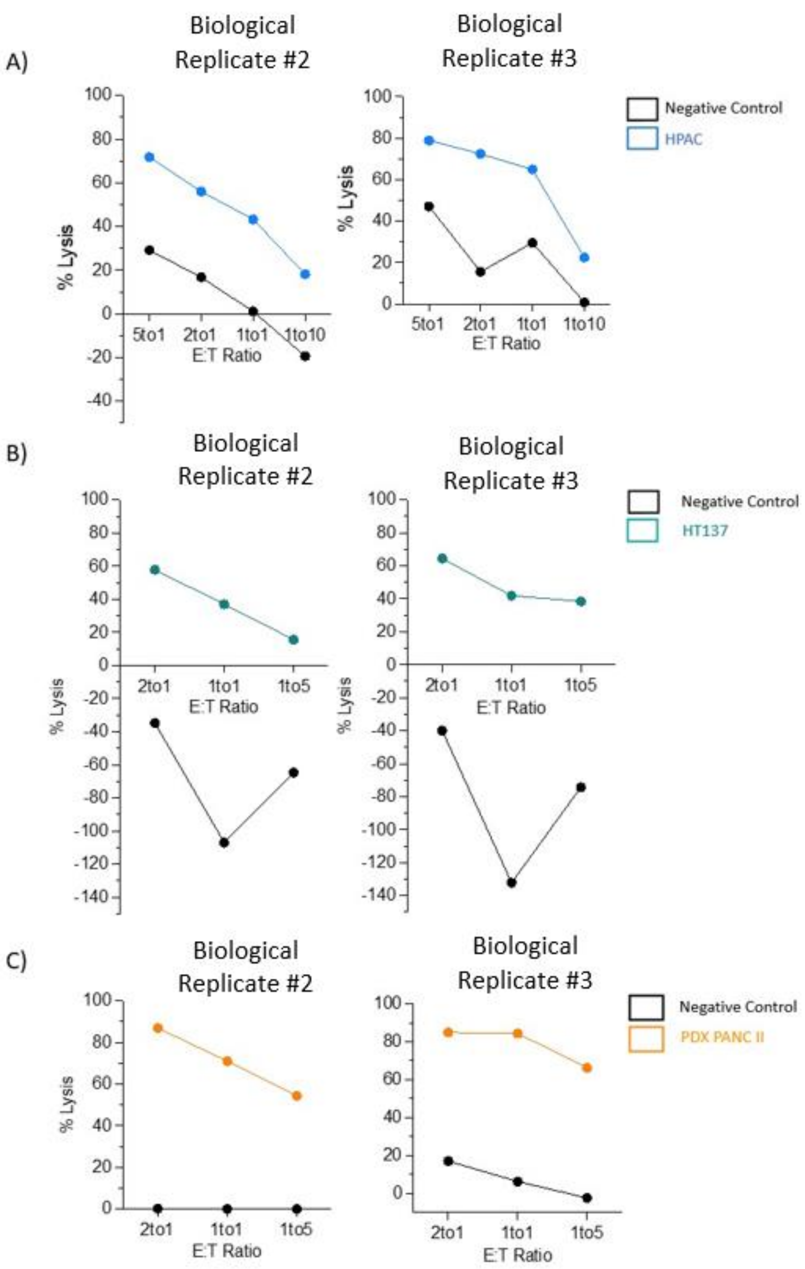

Figure S2. Replicates of cytotoxicity assays that are shown in Figure 3 of A) HPAC, B) HT137, and C) PANCII PDX cells. Negative values represent tumor growth compared to controls. CAR T cells derived from two additional healthy donor PBMCs as biological replicates. Negative control represents coculture with a Muc16CD negative cell line (either 3T3-GFPLuc or Panc1-GFPLuc cells).

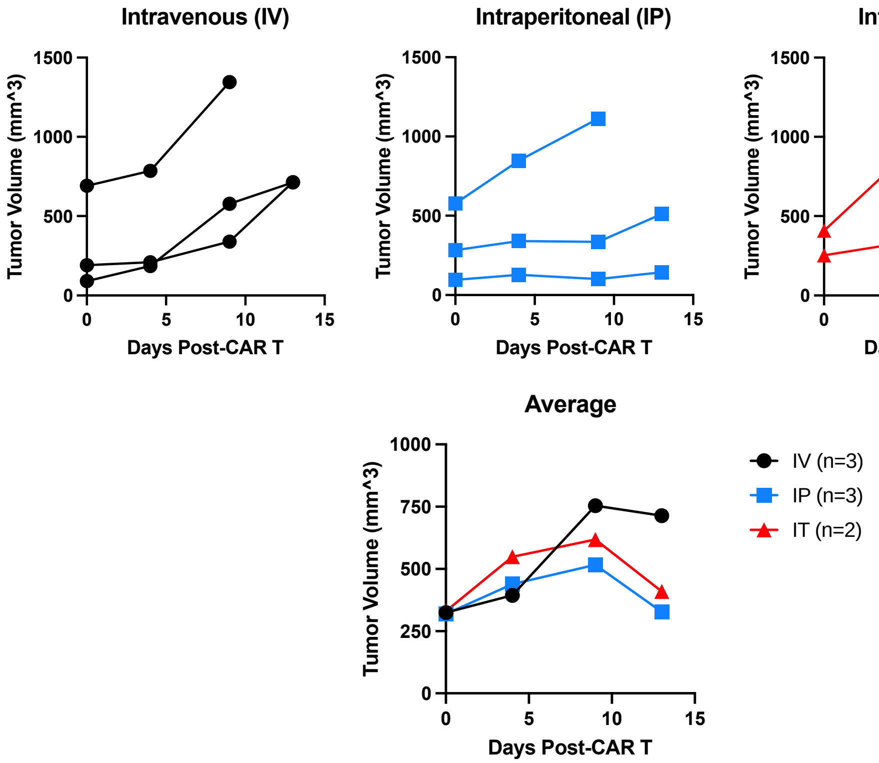

Figure S3. Intravenous and intraperitoneal administration of CAR T cells similarly affects tumor growth kinetics. Mice were engrafted with 1E6 Panc1-Muc16CD tumor cells and then treated with 2E6 CAR T cells by intravenous, intraperitoneal, or intratumoral administration.
